# The dynamic history of gymnosperm plastomes: Insights from structural characterization, comparative analysis, phylogenomics, and time divergence

**DOI:** 10.1002/tpg2.20130

**Published:** 2021-09-10

**Authors:** Lubna, Sajjad Asaf, Abdul Latif Khan, Rahmatullah Jan, Arif Khan, Adil Khan, Kyung‐Min Kim, In‐Jung Lee

**Affiliations:** ^1^ Dep. of Botany, Garden Campus Abdul Wali Khan Univ. Mardan 23200 Pakistan; ^2^ Natural and Medical Sciences Research Center Univ. of Nizwa Nizwa 616 Oman; ^3^ Dep. of Biotechnology, College of Technology Univ. of Houston Houston TX 77204 USA; ^4^ Division of Plant Biosciences, School of Applied Biosciences, College of Agriculture & Life Science Kyungpook National Univ. Daegu 41566 Republic of Korea; ^5^ Genomics Group Faculty of Biosciences and Aquaculture Nord Univ. Bodø 8049 Norway; ^6^ Institute of Genomics for Crop Abiotic Stress Tolerance, Dep. of Plant and Soil Science Texas Tech Univ. Lubbock TX 79409 USA

## Abstract

Gymnosperms are among the most endangered groups of plant species; they include ginkgo, pines (Conifers I), cupressophytes (Conifers II), cycads, and gnetophytes. The relationships among the five extant gymnosperm groups remain equivocal. We analyzed 167 available gymnosperm plastomes and investigated their diversity and phylogeny. We found that plastome size, structure, and gene order were highly variable in the five gymnosperm groups, of which *Parasitaxus usta* (Vieill.) de Laub. and *Macrozamia mountperriensis* F.M.Bailey had the smallest and largest plastomes, respectively. The inverted repeats (IRs) of the five groups were shown to have evolved through distinctive evolutionary scenarios. The IRs have been lost in all conifers but retained in cycads and gnetophytes. A positive association between simple sequence repeat (SSR) abundance and plastome size was observed, and the SSRs with the most variation were found in Pinaceae. Furthermore, the number of repeats was negatively correlated with IR length; thus, the highest number of repeats was detected in Conifers I and II, in which the IRs had been lost. We constructed a phylogeny based on 29 shared genes from 167 plastomes. With the plastome tree and 13 calibrations, we estimated the tree height between present‐day angiosperms and gymnosperms to be ∼380 million years ago (mya). The placement of Gnetales in the tree agreed with the Gnetales–other gymnosperms hypothesis. The divergence between *Ginkgo* and cycads was estimated as ∼284 mya; the crown age of the cycads was 251 mya. Our time‐calibrated plastid‐based phylogenomic tree provides a framework for comparative studies of gymnosperm evolution.

## Core Ideas

- We analyzed 167 available gymnosperm plastomes and investigated their diversity and phylogeny.
- The plastomes vary in genome architecture, size, gene order, SSR, and inverted repeat evolution.
- The tree height between present day angiosperms and gymnosperms was 380 mya.
- The divergence between *Ginkgo* and cycads was estimated as ∼284 mya.

**Abbreviations**
cpSSRs: chloroplast simple sequence repeats
GC: guanine–cytosine
IR: inverted repeat
mya: million years ago
SSR: simple sequence repeats

## 1 INTRODUCTION

Gymnosperms (a group of seed‐bearing plants) are found on all continents except Antarctica; two‐thirds of the gymnosperms are conifers, a group that makes up more than 39% of the world's forests (Armenise et al., 2012). Gymnosperms play important roles in the global carbon cycle; contribute to reducing soil erosion; and provide valuable sources of wood, resin, medicine, and food (Zonneveld, 2012; Murray, 2013). Overall, the group consists of 12 families with 83 genera and around 1,079 species (Christenhusz & Byng, 2016). Unlike the seeds of flowering plants (i.e., angiosperms), those of gymnosperms grow on scales or leaf surfaces; they are not enclosed within an ovary and are known as “naked seeds”. According to molecular data, the gymnosperms can be classified into five classes: Conifers I (pines), Conifers II (cupressophytes), ginkgo, cycads, and gnetophytes (Rai et al., 2008, Chaw et al., 2018).

As the largest lineage of gymnosperms, the conifers were divided into seven families by Pilger (1926). Two useful books on conifers have been published in the last decade by Eckenwalder (2009) and Farjon (2010), in which 546 and 615 species were identified, respectively. Currently, it is largely accepted that conifers contain two major clades, Pinaceae and the remaining non‐Pinaceae conifers (Conifers II or cupressophytes), in which the first and second largest families are Pinaceae and Podocarpaceae (Knopf et al., 2012). Overall, the cupressophytes include five families, namely the Cupressaceae, Araucariaceae, Sciadopityaceae, Taxaceae, and Podocarpaceae, with approximately 405 species (Gernandt et al., 2011). The gnetophytes contain three families (Gnetaceae, Ephedraceae, and Welwitschiaceae), each of which contains a single genus. The division of the 10 cycad genera into two families (Zamiaceae and Cycadaceae) was recently confirmed by molecular phylogenetic studies (Chaw et al., 2005; Zgurski et al., 2008; Salas‐Leiva et al., 2013).

The chloroplasts of plants, which are considered to have been derived from ancient endosymbiosis with cyanobacteria, retain their own unique DNA that encodes multiple genes, including components of the light reaction pathways in photosynthesis that convert light energy into chemical energy (Schimper, 1883, Martin et al., 2002). Indeed, photosynthesis is strictly regulated by genes in the chloroplasts (Soll & Schleiff, 2004). Many nonessential genes have been lost in the chloroplast, whereas some functional genes have been transferred to the nuclear genome (Soll & Schleiff, 2004). Various researchers have previously published on gene transfer from the chloroplast to the nucleus and its potential causes (Martin & Herrmann, 1998; Martin, 2003). Chloroplast genome size, genome composition, and gene number can differ in gymnosperms even more than in angiosperms because the former have diverse evolutionary histories and genetic origins (Moore et al., 2007; Chaw et al., 2018). In nearly all the main lineages of plants, the small shift in chloroplast genome size suggests that the genome has been preserved by natural selection, especially compared with the spontaneous and large‐scale changes seen in both mitochondrial and nuclear genomes (Alexeyev et al., 2004; Greilhuber et al., 2005).

**Core Ideas**

- We analyzed 167 available gymnosperm plastomes and investigated their diversity and phylogeny.
- The plastomes vary in genome architecture, size, gene order, SSR, and inverted repeat evolution.
- The tree height between present day angiosperms and gymnosperms was 380 mya.
- The divergence between *Ginkgo* and cycads was estimated as ∼284 mya.

**FIGURE 1:**
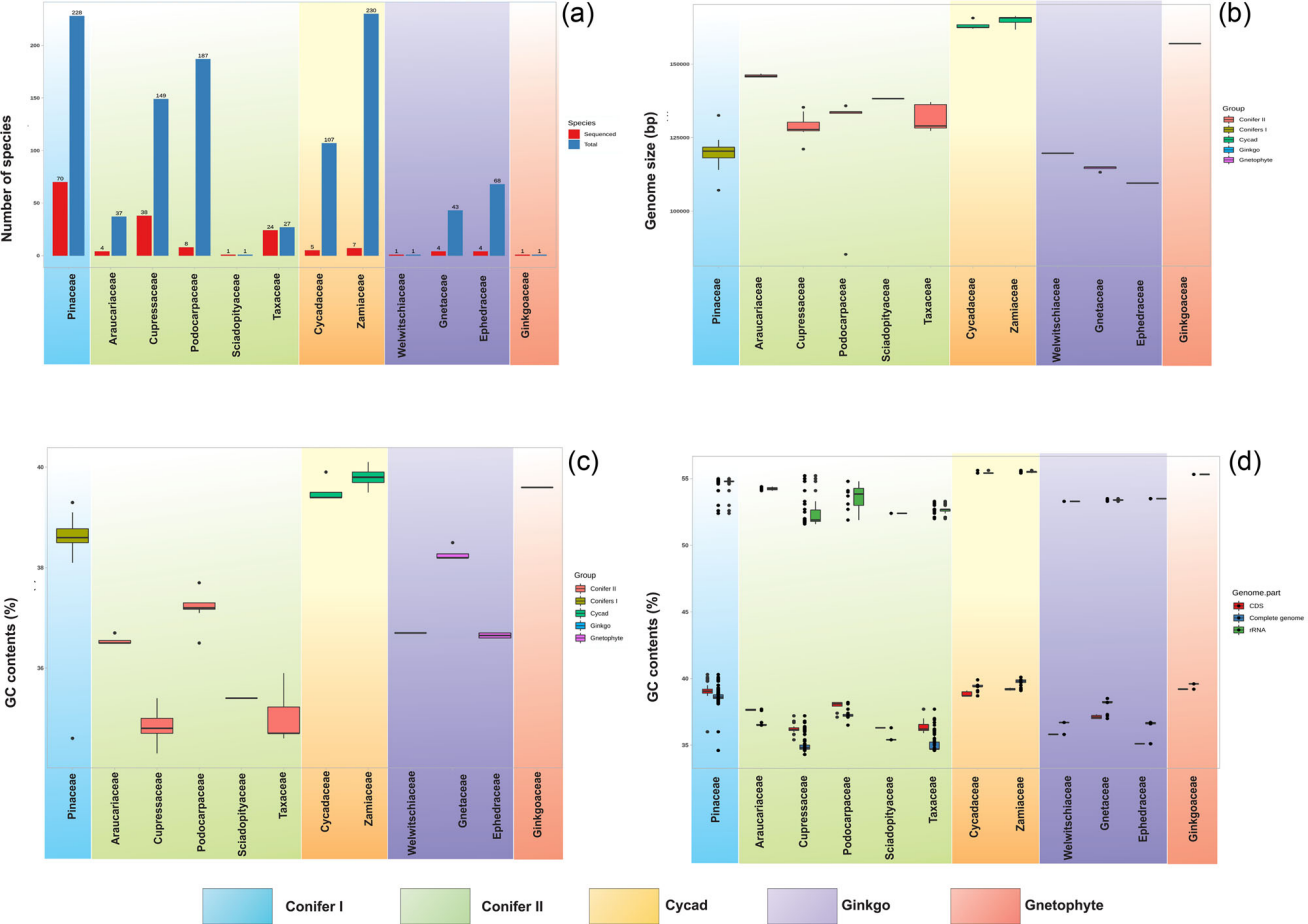
(a) Bar plot showing gymnosperm plastomes publicly available on GenBank. The red bar shows sequenced plastomes; the blue bar shows total number of plastomes. (b) A summary of plastome size. (c) Guanine–cytosine (GC) contents across gymnosperms. (d) The GC contents (%) in different parts of gymnosperm plastomes

In 1994, the first complete *Pinus thunbergii* Parl. (black pine) gymnosperm plastome was sequenced (Wakasugi et al., 1994). With the introduction of next‐generation sequencing technology, efforts to decode the complete plastome of plants have increased. About 167 gymnosperm plastomes, including plastomes from all 12 recognized families, were available from GenBank on 12 June 2020 (Figure 1). In previous studies on seed plants, three significant factors have been suggested as driving the variation in chloroplast genome size: (a) intergenic region variation, which primarily affects variation in the size of the chloroplast genome within a genus (Masood et al., 2004; Wu et al., 2007); (b) variation in an inverted repeats (IRs) region, which is an important feature of specific groups; and (c) gene loss, which is a significant cause of certain parasitic plants reducing the size of their chloroplast genome (Wolfe et al., 1992; Wakasugi et al., 1994; Qu et al., 2019).

The question of which of these three factors has the greatest impact on genome size remains unclear. The contribution of natural selection to genome variation has yet to be fully elucidated. Indeed, previous analyses of gymnosperm plastomes have yielded generally unclear findings and there remains a lack of detailed systematic phylogenetic studies because of low levels of taxon sampling or the inability to make comparisons with distant relatives. Despite considerable effort to determine gymnosperm plastomes at the genus or species level, certain families remain poorly sampled. For example, only 10 of the 29 Cupressaceae genera and 4 of the 19 Podocarpaceae genera have plastomes available (Figure 1a). In fact, with the exception of the monotypic Welwitschiaceae, Sciadopityaceae, and Ginkgoaceae families, none of the gymnosperm families have had >50% of their species sequenced (Figure 1a). For example, only 70 of the 228 Pinaceae species, four of the 37 Araucariaceae species, and eight of the 187 Podocarpaceae species have been sequenced. Furthermore, sampling bias is evident in the sequencing of these families, and several genera do not have a single GenBank representative. In the past decade, the plastomic characteristics of land plants (Wicke et al., 2011; Daniell et al., 2016), ferns (Wolf et al., 2011), seed plants (Jansen & Ruhlman, 2012), and flowering plants (Ruhlman & Jansen, 2014) have all been investigated; nevertheless, an comprehensive study of plastome evolution in gymnosperms is lacking.

Therefore, in the present study, 167 complete gymnosperm plastomes available in the NCBI database (12 June 2020) were downloaded and annotated. The aim was to use our analyses to address the essential questions related to gymnosperm plastomes as follows: (a) the general structures of gymnosperm plastomes, including gene order, gene content, gene gain, gene loss, and variance in genome size; (b) how different sequence characteristics influence gymnosperm plastome size variations; (c) how IR regions evolved in gymnosperm plastomes; (d) which simple sequence repeats (SSRs) exist in the plastomes and whether variation exists in repeat sequences; (e) the hotspot divergence regions and genes potentially under positive selection; and (f) whether we can identify the gymnosperm phylogenetic relationships and divergence of the major gymnosperm lineages based on the genes shared among the plastomes.

## 2 MATERIALS AND METHODS

### 2.1 Taxon sampling

The complete plastomes of 167 gymnosperm species available in GenBank (as of May 18, 2020) were downloaded from the NCBI database (https://www.ncbi.nlm.nih.gov/genome). The species with incorrect annotations were reannotated by CpGAVAS (Liu et al., 2012) and DOGMA (Wyman et al., 2004) (http://dogma.ccbb.utexas.edu/). Moreover, tRNAscan‐SE version 1.21 (Lowe & Eddy, 1997) was used to detect tRNA genes. Finally, the annotations were verified by Geneious Prime (Kearse et al., 2012). We used Circos 0.68 (Krzywinski et al., 2009) to draw plastome maps with their different characteristics. The details (species name, family name, order name, genome size, and accession number) of the 167 plastomes are listed in Supplementary Table S1. Graphical representations were created in R 4.0 and the ggplot2 package (Wickham, 2009). The number of shared genes among Conifer II families was identified by the Venn diagram webtool (bioinformatics.psb.ugent.be/webtools/Venn/).

### 2.2 Characterization of repetitive sequences and SSRs

REPuter was used to determine the repetitive sequences (direct, reverse, and palindromic repeats) within plastomes (Kurtz et al., 2001). For repeat identification, the following settings were used in REPuter: (a) A minimum repeat size of 30 bp, (b) ≥90% sequence identity, and (c) a Hamming distance of 1. Tandem Repeats Finder version 4.07b was used to find tandem repeats with the default settings applied (Benson, 1999). To find SSRs, MISA (Beier et al., 2017) was used with the search parameters set to ≥3 repeat units for pentanucleotide and hexanucleotide repeats, ≥4 repeat units for trinucleotide and tetranucleotide repeats, ≥8 repeat units for dinucleotide repeats, and ≥10 repeat units for mononucleotide repeats.

### 2.3 Phylogenetic analyses and divergence time

To resolve the phylogenetic position of gymnosperms, 29 shared genes from 167 plastomes were used for the analysis. Initially, a separate maximum likelihood analysis of these data was conducted using RAxML (Stamatakis et al., 2008) implemented in CIPRES with the default general time reversible model and the fast bootstrap option previously reported by Crisp and Cook (2011). The resulting phylogenetic reconstruction was displayed using FigTree version 1.4.1 (Rambaut, 2009) and Interactive Tree Of Life, Version 6 (Letunic & Bork, 2019).

We used a concatenated data matrix to determine the divergence time of gymnosperms relative to those of four angiosperm species, Briefly, the default general time reversible substitution model was used with four rate categories. A Yule tree speciation model was applied with a log‐normal relaxed clock model in BEAST (Bouckaert et al., 2014) with a prior rate of substitution. We used an average substitution rate of 3.0 × 10^−9^ substitutions per site per year and a fossil‐based method to calibrate the molecular divergence. To root the calibration time, we included four outgroup angiosperm species: *Magnolia denudata* Desr., *Acorus gramineus* Aiton, *Platanus occidentalis* L., and *Amborella trichopoda* Baill. We also incorporated 13 fossil constraints (Supplementary Table S2) that are widely recognized and have been used previously for molecular dating of gymnosperms or seed plants (Crisp & Cook, 2011); almost every main lineage of gymnosperm was calibrated by at least one fossil record (Supplemental Table S2). The mean root height constraint of 355 million years ago (mya) was based on the work of Won and Renner (2006), who assigned minimum and maximum ages of 325 and 385 mya, respectively, on the basis of fossils with fused (shared by all extant seed plants) and unfused integuments, respectively. This calibration is consistent with the review by Sanderson et al. (2004) and previous fossil‐constrained estimates (Magallóan & Sanderson, 2005; Smith et al., 2010). The calibration limited to the root height was used to compare the internal calibrations and to assess their influence on divergence time estimates. The dating analyses involved three independent Markov chain Monte Carlo runs of 25 million generations. LogCombiner (http://beast.bio.ed.ac.uk/LogCombiner) was used to combine the tree files from each of the three runs. Convergence and effective sample sizes were assessed in Tracer 1.5 (Rambaut et al., 2018). From each analysis, we removed 25% of the trees as burn‐in. Finally, the tree was calculated by TreeAnnotator (https://www.beast2.org/treeannotator/) and the tree with the 95% highest posterior density was visualized in FigTree 1.4 (http://beast.bio.ed.ac.uk/FigTree).

## 3 Results

### 3.1 Gymnosperm plastome characteristics (genome size and guanine–cytosine content)

Gymnosperm plastomes were highly variable in size, ranging from 85,318 [Conifers II: *Parasitaxus usta* (Vieill.) de Laub.] to 166,341 bp (cycad: *Macrozamia mountperriensis* F.M.Bailey), with a mean of 128,080 bp (Figure 1b). The largest plastomes were those of cycads, ranging from 161,815 (*Dioon spinulosum* Dyer ex Eichl.) to 166,341 bp, followed by ginkgo (156,988 bp in *Ginkgo biloba* L.). The size of gnetophytes ranged from 109,518 (*Ephedra equisetina* Bunge) to 119,726 bp (*Welwitschia mirabilis* Hook.f.). However, size variation was greatest in both Conifers I and II groups: from 107,122 (*Cathaya argyrophylla* Chun & Kuang) to 132,588 bp (*Taiwania cryptomerioides* Hayata) in Conifers I and from 85,318 to 146,723 bp (*Araucaria heterophylla* (Salisb.) Franco) in Conifers II. Similarly, the guanine–cytosine (GC) content of gymnosperm plastomes varied considerably from 34.3% (*Thuja* spp.) to 40.11% (*Dioon spinulosum*) (Figure 1c). In Conifers I, huge variation was observed in GC content (34.6–39.3%), whereas GC content was less variable in Conifers II (34.3–37.7%) and gnetophytes (36.6–38.5%). The highest and most consistent GC content was detected in cycads at 39.4–40.1% (Figure 1c). Similar to angiosperm plastomes, the nucleotide composition of gymnosperm plastomes was overwhelmingly GC‐poor; however, GC content was not evenly distributed across the gymnosperm plastomes. In all the gymnosperm groups, the highest GC percentage was observed in rRNA relative to other parts of the plastome; the highest GC content in rRNA was found in the Cycadaceae and Zamiaceae (55.6%); the lowest was found in the Cupressaceae (51.6%) (Figure 1d).

### 3.2 Gene content and gene loss in gymnosperm plastomes

**FIGURE 2:**
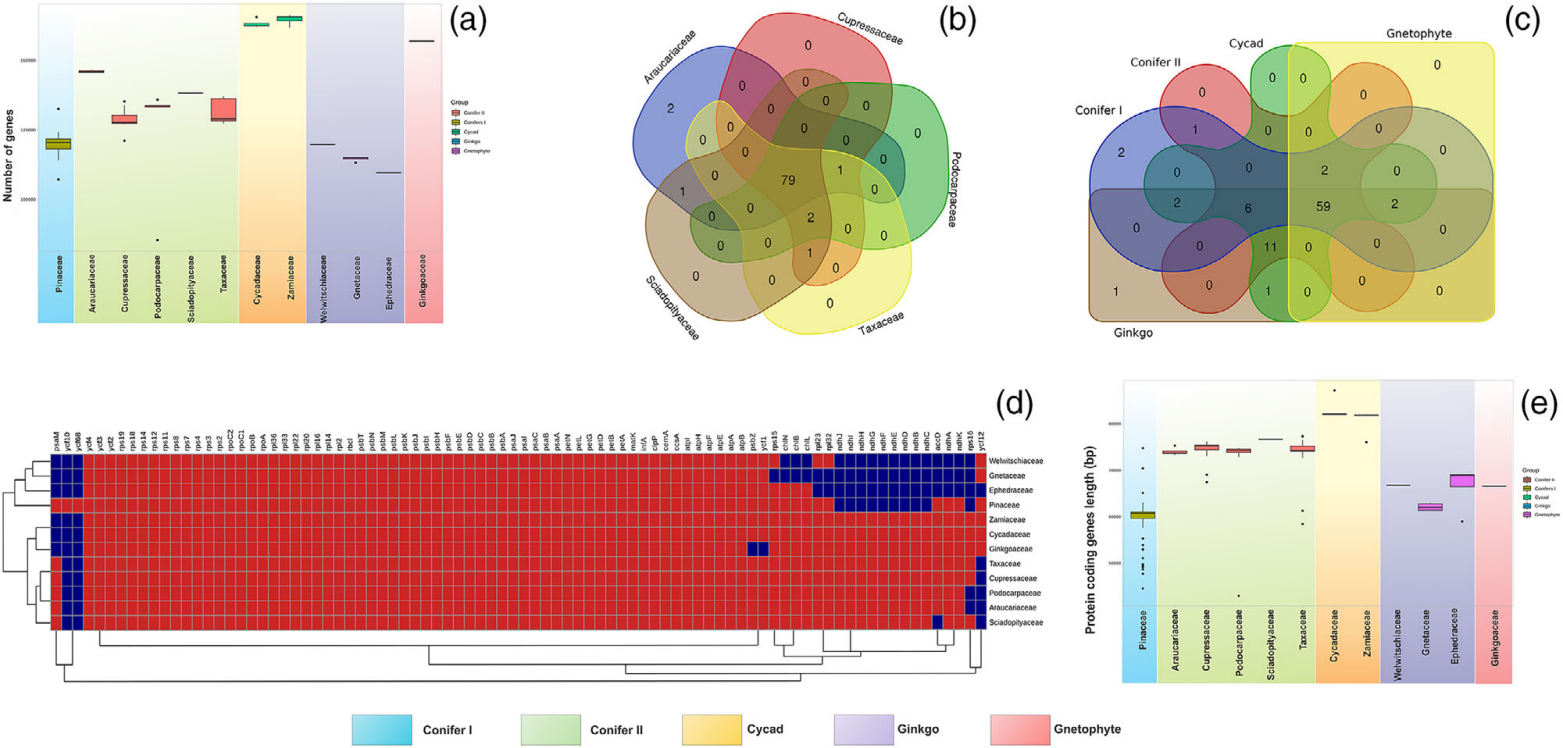
(a) Number of genes across gymnosperm plastomes, (b) number of shared genes among Conifer II families, (c) number of shared genes among five gymnosperm groups’ plastomes, (d) summary of genes loss across gymnosperm plastomes, and (e) the length of protein coding genes (bp) in gymnosperm plastomes

The plastomes of the five gymnosperm groups had 37 to 99 protein‐coding genes, 4 to 8 rRNA genes, and 15 to 40 tRNA genes. The total number of genes in a plastome varied from 76 (*Parasitaxus usta*) to 137 (*Pinus taiwanensis* Hayata) (Figure 2a). Analysis revealed that the loss of an IR copy resulted in the removal of about 14 genes from Pinaceae. Similarly, the Ephedraceae, Welwitschiaceae, and Gnetaceae lost all *ndh* genes (Figure 2b). About 67 genes were shared in Conifers I, whereas 59 were shared by all five gymnosperm groups (excluding the *Parasitaxus usta* plastome) (Figure 2c,d). Among the cycad plastomes, the gene content was highly conserved, with some exceptions in *Stangeria eriopus* (Kunze) Baill., in which *chlB, chlL, chlN, psaJ, psaM*, and *rpl23* were lost or pseudogenized, and *trnT* ^GGU^ was completely lost (Figure 2b). Similarly, *rpl23* was pseudogenized in both *Cycas szechuanensis* C.Y.Cheng, W.C.Cheng & L.K.Fu and *Ginkgo* plastomes, and lost in Ephedraceae and Gnetaceae plastomes. In gnetophytes, variations in plastid gene content were mostly a result of IR contraction or expansion. For example, the genes *chlB, chlL, chlN, ndh* (11 genes), *accD*, *psaM, rpsl23, rpl32, rps15*, and *rps16* were lost from the Gnetaceae through the contraction of an IR region. With the exception of *rps15* and *rpl32*, these genes were also lost in the Welwitschiaceae. Variation was observed in Pinaceae plastomes, and different genes were lost or pseudogenized through IR loss. Interestingly, *Parasitaxus usta* lost nearly 60% of the typical gymnosperm genome coding capacity; it retained only 33, 31, and 4 intact protein‐coding, tRNA, and rRNA genes, respectively, and lost almost all the *ndh, pet, psa, psb, rbcL, atpF, atpI ccsA, cemA, chB, chlL*, and *chlN* genes. Notably, these losses almost exclusively affected photosynthesis genes in the *Parasitaxus usta* plastome, which was the smallest and least functionally capable plastome of all gymnosperms. Furthermore, in Taxaceae plastomes, *rpl33* was lost in *Cephalotaxus* but *clpP* was absent in *Taxus*. In addition, r*ps16* was lost in Cupressaceae plastomes. Moreover, *ycf10*, *ycf12*, and *ycf68* were detected in some species of Pinaceae only. Notably, *Sciadopitys verticillata* (Thunb.) Siebold & Zucc. was the only cupressophyte species in which the plastid *accD* was lost. Similarly, this gene was lost in the Araucariaceae, Sciadopityaceae, Ephedraceae, and Gnetaceae.

Analysis of total protein‐coding length (bp) revealed substantial variation in gymnosperm plastomes, with lengths varying from 42,933 bp in *Parasitaxus usta* (Podocarpaceae) to 87,147 bp in *Cycas revoluta* Thunb. (Cycadaceae). The highest variation was observed in Pinaceae plastomes, in which the lowest length was 44,501 bp in *Picea sitchensis* (Bong.) Carrière and the highest was 74,778 bp in *Taiwania cryptomerioides* (Figure 2e).

### 3.3 Simple sequence repeat analysis in gymnosperm plastomes

**FIGURE 3:**
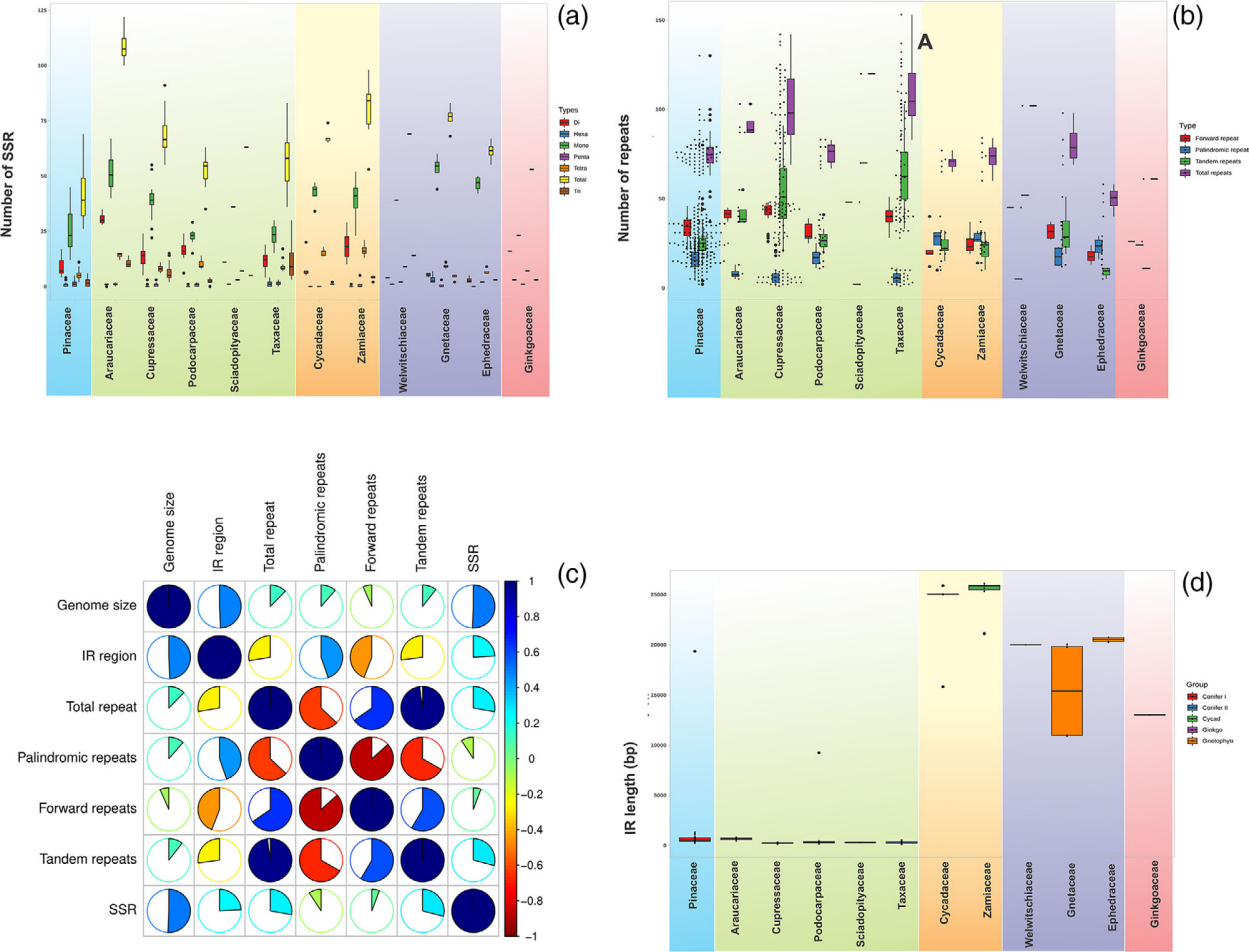
(a) Number of simple sequence repeats (SSR) detected in gymnosperm plastomes, (b) number of functional repeats detected in gymnosperm plastomes, (c) correlation among different characteristics of gymnosperm plastomes, and (d) inverted repeats (IR) length (bp) variation in gymnosperm plastomes

Simple sequence repeats are repeating sequences of typically 1–6 bp that are distributed throughout the genome. In this study, we analyzed perfect SSRs in all the studied plastomes (Figure 3a). Similar to other plastome characteristics, there was great variation in the number of SSRs in gymnosperm plastomes: SSR numbers ranged from 26 (*Pinus pinea* L.) to 122 [*Agathis dammara* (Lamb.) Rich. & A.Rich.]. Surprisingly, the lowest number of SSRs was detected in Conifers I but the highest was in Conifers II, followed by cycads (Figure 3a). The highest number of SSRs was found in Araucariaceae plastomes (100–122), and the most variable SSRs were detected in Pinaceae (26–69) and followed by Taxaceae (36–83) plastomes. Here, we observed a positive association between SSR numbers and plastome size in conifers. The second most abundant SSR count was observed in the family Zamiaceae, suggesting the positive relationship between plastome size and GC content. In all plastomes, the most abundant repeat motifs were mononucleotides, ranging from 12 in *Picea abies* (L.) H.Karst. to 67 in *Agathis dammara*, followed by dinucleotides, which were the second most abundant in all families except the Taxaceae (in which trinucleotides were the second most common repeats) (Figure 3a). According to our search criterion, pentanucleotide and hexanucleotide SSRs were found in 104 and 78 plastomes, respectively; however, neither of these motifs were detected in cycad plastomes and only one hexanucleotide was detected in *Lepidozamia peroffskyana* Regel from the Zamiaceae. Similarly, pentanucleotide motifs was only detected in three Zamiaceae plastomes. Hexanucleotide SSRs were also absent in Ephedraceae plastomes.

### 3.4 Functional repeats within gymnosperm plastomes

Repeat analysis of 167 plastomes revealed a highly variable number of palindromic, forward, and tandem repeats. Large IRs play important roles in maintaining a conserved arrangement and stabilizing plastomes. The complete IR was lost in the plastomes of both Conifers I and II; therefore, many more rearrangements were detected in these plastome than in those of higher plants. The total number of repeats (including palindromic, forward, and tandem repeats) in these genomes ranged from 37 (*Picea koraiensis* Nakai) to 153 (*Cycas hainanensis* C.J.Chen ex C.Y.Cheng, W.C.Cheng & L.K.Fu). The highest number of repeats was found in Conifers II, in which the number ranged from 67 (*Parasitaxus usta*) to 153 (*Cycas hainanensis*), and particularly in the Cupressaceae (92–142) and Taxaceae (83–153) (Figure 3b). Among these repeats, the highest number of palindromic repeats was detected in the cycads (10–32) followed by the Ephedraceae. In contrast, the lowest number of palindromic repeats was detected in conifers II, especially in Cupressaceae and Taxaceae. The highest number of forward repeats was found in Conifers II, especially in the Cupressaceae (26–48), followed by some Conifer I plastomes. Similarly, the highest number of tandem repeats was detected in Conifers II, particularly in the Cupressaceae and Taxaceae (19–103). Overall, there was a negative correlation between the number of repeats and IR length in the plastomes (Figure 3c). Typically, the highest number of repeats and rearrangements were detected in Conifers I and II, in which complete IRs had been lost.

### 3.5 Evolution of IRs in gymnosperm plastomes

**FIGURE 4:**
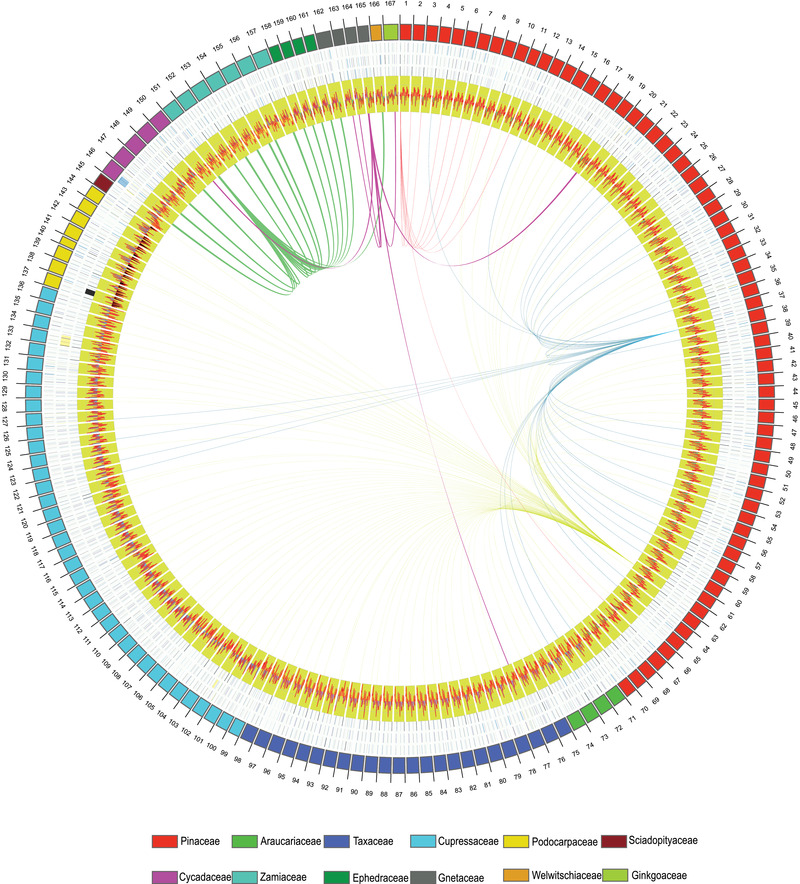
Circos view of the 167 gymnosperm plastomes (from five gymnosperm groups), shown in different colors. The tracks from outside to inside are coding sequences, tRNA, rRNA, gene length(heatmap), guanine–cytosine (GC) skew. Inverted repeats (IR) are linked by lines in different colors: IR > 20 kb (green), IR between 10 and 20 kb (pink), IR >9 kb (red), IR > 500 bp (blue), and IR < 500 bp (yellow). Numbers 1–167 shows the corresponding gymnosperm plastomes (detailed in Supplementary Table S1)

Among the five groups of gymnosperms, plastome structure was variable. With a pair of large IRs, differentiated by a large single‐copy region and a small single‐copy region, the structure was quadripartite in ginkgo, gnetophytes, and cycads. The IR can be identified by a central unit of four rRNA genes (i.e., *rrn4.5*, *rrn5*, *rrn16*, and *rrn23*). Comparative analyses of the plastomes suggested that Conifers I and II had lost their IRs, although the largest IR regions were found in *Nothotsuga longibracteata* (W.C.Cheng) H.H.Hu ex C.N.Page (19,255 bp) followed by *Parasitaxus usta* (9,246 bp). As Conifers I and II had both lost IR regions, however, several differences were observed, such as all *ndh* genes being lost or pseudogenized in Pinaceae and retained in Conifers II. The largest IR regions were found in cycad members, in which the IR length was >20 kb (except in *Cycas taitungensis*, in which the IR was 15,830 bp in length) (Figure 3d). For example, the largest IR region was found in *Ceratozamia hildae* G.P.Landry & M.C.Wilson (26,137 bp in length) followed by *Lepidozamia peroffskyana* and *Macrozamia mountperriensis* (25,918 bp). Gnetophytes had the next largest IR regions, which ranged from 10,879 bp (*Gnetum montanum* Markgr.) to 20,743 bp (*Ephedra sinica* Stapf.) in length; the largest IR regions were found in the Ephedraceae relative to the Gnetaceae (Figure 3d). Surprisingly, a large IR (19,355 bp in length) was detected in the *Nothotsuga longibracteata* plastome from the Pinaceae. A comparison of IRs also revealed that those in cycads are evolutionarily conserved and static. However, *Ginkgo* IR regions showed contraction, with the exclusion of some genes such as *ycf2*. The distribution of IRs across gymnosperm plastomes was illustrated with Circos software (http://circos.ca/) (Figure 4).

### 3.6 Plastomes’ phylogenomics and diversification of gymnosperms

**FIGURE 5:**
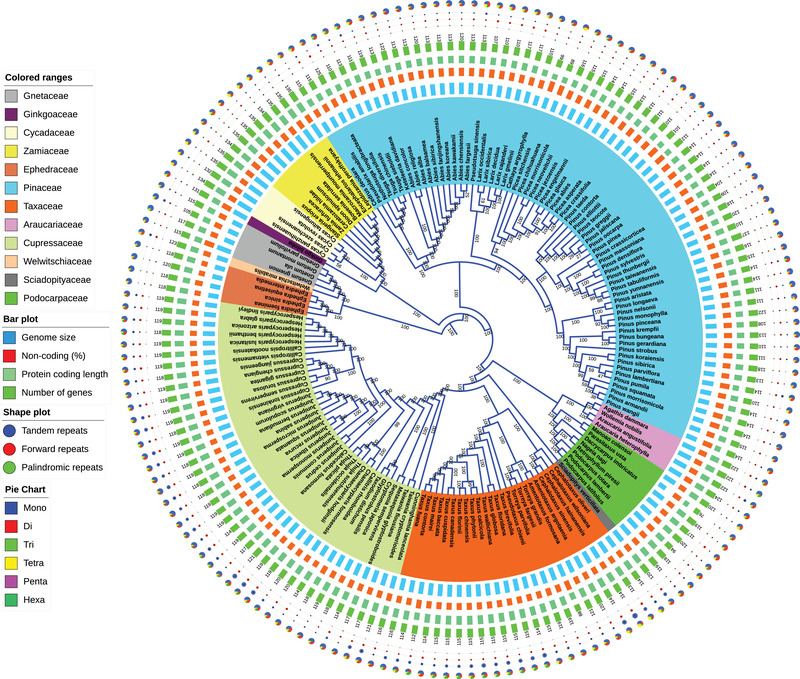
Maximum likelihood phylogenetic reconstruction of gymnosperms based on 29 shared genes among 167 plastomes. The distribution of genome sizes, noncoding genes (%), protein coding lengths, and the number of genes are shown in blue, red, turquoise, and green bar plot, respectively. The shape plot shows tandem repeats (dark blue), forward repeats (red), and palindromic repeats (green). The pie chart shows the distribution of simple sequence repeats (SSRs) across gymnosperm plastomes. Number above the branches are the bootstrap values of 1,000 replicates

Since the early 20^th^ century, the phylogenetic relationships among the five main classes of living gymnosperms have been fiercely debated (Chaw et al., 2018). To provide further insights into these relationships, we used 29 protein‐coding genes shared by all gymnosperm plastomes to construct phylogenetic trees. Our study therefore provides the first molecular phylogeny of gymnosperms based on shared genes from all the gymnosperm species available in the NCBI database. The extant angiosperms used as an outgroup formed a monophyletic group in the trees. Similarly, the gymnosperms formed a monophyletic group. The phylogenetic position of Gnetales has remained in question in plant science; our phylogenetic trees indicated that Gnetales was more closely related to angiosperms than to the Coniferales (Figure 5). However, both *Ginko* and Cycadales were positioned in a similar manner to previous studies: They were closely related, either as sister groups or as a clade. As the Pinaceae were a sister group to all Coniferales, *Abies* formed a close clade with *Kateleeria*, whereas *Nothotsuga* and *Pseudolarix* formed clades with *Tsuga*. *Cathaya* was not closely related to any other genera within the Pinaceae. In Conifers II, the Sciadopityaceae were not closely related to any genera; however, they were situated between the Araucariaceae and Taxaceae.

**FIGURE 6:**
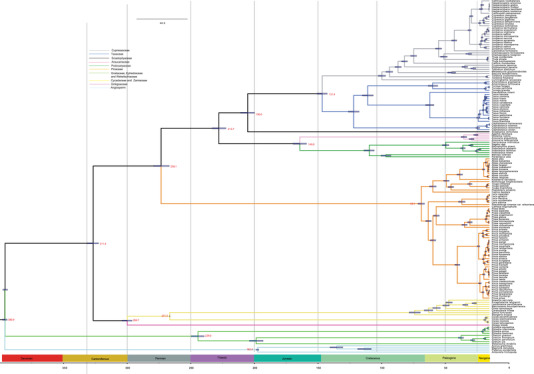
Maximum credible molecular chronogram (time tree) of gymnosperms from BEAST, with branch lengths proportional to time and log‐normal fossil‐based calibrations. Different colored branches represent gymnosperm families. The 95% highest posterior density credibility intervals are shown for the node ages (million years ago, mya). A geological time scale is shown at the bottom

To obtain the secondary calibration points and topology information for the 167 gymnosperms, we performed a phylogentic reconstruction and divergence time estimation by using the 29 protein‐coding genes shared among the plastomes. The tree topology resulting from BEAST analysis suggested that a sister relationship existed between ginkgo and the cycads. The divergence between ginkgo and the cycads was estimated to have occurred ∼284 mya, whereas the crown age of the cycads was estimated at 251 mya according to the speciation birth–death model (Figure 6). We estimated the tree height (group age between present‐day angiosperms and gymnosperms) to be 380 mya, which is similar to previously reported estimates (360 or 330 mya) (Clarke et al., 2011).

## 4 DISCUSSION

In this study, 167 available plastomes from five gymnosperm groups were analyzed. Plastome size, structure, and gene order were found to be highly variable, as previously reported (Parks et al., 2009; Asaf et al., 2018). The variation in gymnosperm plastome size may have been caused by a number of factors. The first factor is the loss of IR regions, which has largely reduced the conifers’ plastome size. The second factor is the absence of essential genes, intergenic spacer reduction, and the lack of introns detected in gnetophytes (McCoy et al., 2008; Chaw et al., 2018). The third factor is the deletion of the *ycf2* gene in the ginkgo IR region, which accounts for around 7 kb and results in a plastome that is smaller than those of cycads (Lin et al., 2012). Therefore, it is reasonable to assume that because of the unstable genome arrangement (Wu et al., 2009) or the lack of IRs or genes (Lin et al., 2012), the size of plastomes differed substantially among the gymnosperms. The fourth factor relates to *Parasitaxus usta*, which has the smallest plastome of the gymnosperms because it has lost almost 60% of its typical coding capacity and retains only 33 protein‐coding genes. Previously, *Cathaya argyrophylla* was reported to have the smallest gymnosperm plastome as a result of losing the IR region, 11 plastid *ndh* genes, and a fragment flanked by *ycf2* and *trn*VGAC (Lin et al., 2010); this could be the fifth factor affecting genome variation. Various mechanisms have been proposed as underlying factors in the evolution of plastome size in gymnosperms. For example, Wu and Chaw (2014) stated that the mutation rate and plastome rearrangement can affect the size of cupressophyte plastomes. Our comparative analysis revealed that only the Gnetaceae and cupressophytes had compact plastomes. Similarly, it was previously reported that gnetophytes and Cupressaceae could have small and compact plastomes because of the effective use of crude DNA and selection for rapid replication (McCoy et al., 2008; Wu et al., 2009; Wu & Chaw, 2016). In addition, Wu and Chaw (2016) identified that substitution rates were inversely related to noncoding material and plastome size. These findings clearly indicate that the plastomic reduction in cupressophytes was driven by accelerated substitution rates and reduced noncoding content, which may be the sixth reason for plastome variation.

Guanine–cytosine content distribution also depends on the plastomic architecture. Among the five studied gymnosperm groups, the highest GC content was detected in the cycads, similar to previous reports (Wu & Chaw, 2015; Jiang et al., 2016). The lowest GC content was detected in *Thuja* plastomes. The bias in GC content is related to two factors. First, each of the IR copies contains four rRNA genes. Second, GC‐biased gene conversion acts more frequently in IRs than large single‐copy and small single‐copy regions. Consequently, elevated GC content is observed in IRs but not in other regions (Wu & Chaw, 2015; Yang et al., 2020). Huge variation in GC content was observed in Conifer I plastomes (34.6–39.3%), which may have been caused by the presence and absence of IR regions in the subgenera *Pinus* and *Strobus*, as previously reported in Pinaceae (Zeb et al., 2019). Similar to previous findings, GC content was not evenly distributed across the plastomes, with the highest GC content being observed in rRNA sections (Chen et al., 2015; Yap et al., 2015; Asaf et al., 2018; Chaw et al., 2018).

Multiple factors also contributed to the variation in plastome gene contents. A major factor is the loss of an IR copy, which has resulted in the removal of about 14 genes from the Pinaceae. However, gnetophytes (Ephedraceae, Welwitschiaceae, and Gnetaceae) have retained their IR regions but lost 11 *ndh* genes, similar to the Pinaceae (Chaw et al., 2018). Chaw et al. (2018) also reported the loss of 11 *ndh* genes in gnetophytes. The variability in the plastid gene content of gnetophytes is largely a result of the expansion or contraction of IRs. It has been proposed that the common ancestor of gnetophytes encountered a series of IR expansions that included *chlN*, *chlL*, *rpl32*, and *rps15*; subsequently, these genes were lost through IR contraction in the common ancestor of Gnetum and *Welwitschia* (Wu et al., 2009).

Interestingly, *Parasitaxus usta* was found to have the smallest and least functionally capable gymnosperm plastome, with 33 protein‐coding genes. *Parasitaxus usta* has retained chlorophyll but all the photosynthetic genes have been physically or functionally lost, making it heterotrophic (Qu et al., 2019). In cycad plastomes, gene content is highly conserved (Wu et al., 2007; Jiang et al., 2016): *psaJ*, *rpl23, chlB, chlL*, and *chlN* have been lost or pseudogenized only in *Stangeria eriopus* (Wu & Chaw, 2015). Fifteen genes exist in the putative ancestral IRs of gymnosperms (Zhu et al., 2016), all of which have been preserved in the IRs of *Cycas deboensis* R.Br., whereas 13 remain in *Ginkgo biloba*, excluding *ycf2* in IRs. Moreover, *ycf10*, *ycf12*, and *ycf68* were detected in the Cupressaceae and some Pinaceae members (Supplementary Figure S1). Protein‐coding gene loss is uncommon, but the gene content of cupressophytes has been altered. In some species of Cupressaceae and Taxaceae, for example, *rps16* is present, but it is absent from both the Araucariaceae and Podocarpaceae (Yap et al., 2015). Similarly, *accD* has been lost in the Araucariaceae, Sciadopityaceae, Ephedraceae, and Gnetaceae, whereas *Sciadopitys verticillata* is the only cupressophyte species in which plastid *accD* has been lost but might have been functionally complemented by a nuclear counterpart (Li et al., 2016).

The diversity in the SSRs of chloroplast genomes is an attractive research area in plant biology because of their codominant inheritance, high reproducibility, multi‐allelic composition, richness, and ease of detection (Powell et al., 1996; Ranade et al., 2014). Chloroplast SSRs (cpSSRs) are normally small tandem mononucleotide repeats, typically found in the chloroplast genome's noncoding regions, which usually exhibit intraspecific repeat number differences (Provan et al., 2001; Jakobsson et al., 2007). Similar to nuclear microsatellites, some essential and special features of the organelle genome in which they occur have been inherited by cpSSRs. The original discovery of cpSSRs by Powell et al. (1995) was supported by access to the complete chloroplast genomes of six plant species, with the while plastomes being used, including the noncoding parts (introns and intergenic spacers) (Powell et al., 1995; Provan et al., 2001). However, few studies have reported on the SSRs of gymnosperm plastomes (Jiang et al., 2016; Yi et al., 2016; Asaf et al., 2018; Chen et al., 2019). In our study, Conifers II (Araucariaceae) had the highest number of SSRs and Conifers I had the lowest, whereas Pinaceae and Taxaceae plastomes showed the most variation in SSR numbers. In these plastomes, a linear relationship between SSR number and plastome size was detected, especially in the Araucariaceae family. A similar correlation was discovered in a previous analysis of whole genomes of gymnosperms (Ranade et al., 2014). We looked at the genomic organization of these plastomes to find an appropriate justification for the lower SSR abundance in the cycads, despite it having the largest plastomes. We found that these results may be attributed to the existence of IR regions in the cycads, as Morgante et al. (2002) previously indicated that SSRs should be more abundant within single‐copy regions than in IR regions. In all plastomes, mononucleotides were the most common repeat motifs. There was a trend for cpSSRs to be marginally more abundant in conifers, gnetophytes, and cycads. These findings are consistent with previous studies that indicated that SSRs are unequally distributed in plastomes; these results may provide additional evidence for the identification of appropriate molecular markers for intraspecific and interspecific polymorphism detection (Powell et al., 1995; Zeb et al., 2019; Yang et al., 2020). Most of the mononucleotides and dinucleotides in the plastomes were composed of adenine and thymine, which may lead to base composition bias; this result is compatible with our knowledge of other plastomes (Yap et al., 2015, Hao et al., 2016). Our results were also consistent with previous reports that the SSRs in plastomes mainly consist of repeats of polythymine or polyadenine and rarely include tandem repeats of cytosine and guanine (Yap et al., 2015; Hao et al., 2016). The SSRs also contribute to the adenine–thymine richness of the plastomes, which has previously been found in Pinaceae (Chagné et al., 2004; do Nascimento Vieira et al., 2014; Du et al., 2017). Regarding polymorphisms at the intraspecific level, the SSRs found in the studied plastomes could potentially be used as markers for assessing the genetic diversity of wild gymnosperm populations.

Numerous repeats have previously been identified in gymnosperm plastomes (Yi et al., 2013; do Nascimento Vieira et al., 2014), but the mechanisms underlying these tandem repeats remain to be elucidated. However, plastome rearrangement, gene expansion, and gene duplication are known to be associated with such repeats (Hirao et al., 2008; Yi et al., 2013; Li et al., 2018). In our study, the Taxaceae and Cupressaceae in Conifers II had the highest number of repeats. We also found a negative correlation between the number of repeats and IR length; therefore, the highest number of repeats was detected in Conifers I and II, which have lost the IR. In phylogenetic research, repeat sequences that play a role in genome rearrangement are known to be useful (Asaf et al., 2018; Yang et al., 2020). In addition, studies of multiple plastomes have shown that repeat sequences are effective causes of indels and substitutions (Yi et al., 2013). Sequence variation and rearrangement of the plastome can occur through mispairing of the slipped strand and incorrect recombination of repeat sequences (Wu, Wang et al., 2011; Li et al., 2018). The existence of such repeats indicates that the locus is a significant reconfiguration hotspot for the plastome (Hipkins et al., 1995; Powell et al., 1995). Moreover, such repeats are useful for identifying genetic markers for phylogenetic and population studies (Hirao et al., 2008).

Large IRs play a significant role in maintaining the conserved structure and stability of plastomes (Palmer & Thompson, 1982; Zhang et al., 2014). An IR copy was lost in the plastomes of tribes in the legume subfamily Papilionoideae (Lavin et al., 1990) during the evolution of the angiosperms, and plastome rearrangements are more common in these species than in species possessing typical IRs (Hirao et al., 2008). The IRs of the five gymnosperm groups analyzed here were shown to have evolved in an idiosyncratic evolutionary manner according to the publicly available plastomes in GenBank. Variation among the IR regions of groups and families was observed in these gymnosperms. Although the IR regions of cycads and gnetophytes have been retained, entire IRs have been lost in the plastomes of Conifers I and II; thus, the plastomes of the conifer groups have several more rearrangements than most higher plants, as previously reported (Strauss et al., 1988). It has been demonstrated that the residual IR in the plastome varies between the Pinaceae and cupressophytes, which suggests that after splitting from a single ancestor, these two conifer clades lost one IR copy independently in their own evolutionary histories (Wu, Wang, et al., 2011; Wu & Chaw, 2014). Plastome rearrangements were more common in Conifers II than in Conifers I, and the reduced IRs in Conifer II (especially in Cupressaceae and Taxaceae) could be substituted by more specific repeats (Wu, Lin, et al., 2011).

In our study, IR length was negatively correlated with the number of repeats (palindromic, forward, and tandem repeats) in gymnosperms. The effects of potential functional repeats in gymnosperms’ plastome rearrangement mechanisms should be investigated in more detail. Overall, repeats >20 kb long were found in the cycads, whereas IR regions >10 kb long were detected in the gnetophytes and ginkgo. In gnetophytes, the IR boundary changes show that the group has undergone several stages of expansion, inversion, and gene depletion, culminating in different IR boundaries (Wu et al., 2009). In comparison, the plastomes of pinaceous species show a substantially reduced pair of IRs containing only *trnI‐CAU* and *psbA*. However, it is difficult to find evidence of an IR trace in the plastomes of cupressophytes. Two inverted copies of *trnI‐CAU* are, for example, hypothesized to be putative residues of IRs in *Cryptomeria* (Hirao et al., 2008), but such inverted copies are not detected in other cupressophyte genera such as *Nageia* (Wu & Chaw, 2014) and *Podocarpus* (do Nascimento Vieira et al., 2014). Surprisingly, in the plastome of *Nothotsuga longibracteata* from the Pinaceae, an extended IR region was detected. However, IRs were lacking in all conifer plastomes (Raubeson & Jansen, 1992). Comparative plastome analyses have indicated that the Pinaceae and cupressophytes lost their IRs individually (Hao et al., 2016; Wu & Chaw, 2016). In contrast, Yi et al. (2013) indicated that when the existence of plastome isomers was taken into account, it was difficult to clarify which IR copy of the Pinaceae had been lost. More comprehensive data will therefore be required from additional research to examine the evolutionary mechanism of IR loss in conifers. Although cycad IRs are evolutionarily static, the IR of ginkgo comprises only 13 genes; Lin et al. (2012) suggested that elimination of the *ycf2* gene required contraction of the ginkgo IR.

The phylogenetic relationships among the five gymnosperm groups are not entirely resolved and remain somewhat unclear. Since the first molecular research supporting the sister relationship between the existing gymnosperms and angiosperms was performed by Hori et al. (1985) with 5S rRNA sequences, the molecular phylogenies of several gymnosperms have been reported. Although low‐copy nuclear genes and expressed sequenced tags sequences have been used in phylogenetic gymnosperm reconstruction over recent years (Lee et al., 2011; Xi et al., 2013; Lu et al., 2014), cytoplasmic DNA markers and/or nuclear ribosomal DNA (nrDNA) are still used in most studies. Here, we summarize the main achievements in phylogenetic reconstruction. The divergence of gymnosperms and angiosperms might be dated to approximately 300 to 350 mya in the Carboniferous period on the basis of fossil evidence and molecular clock calibration (Hedges et al., 2006; Clarke et al., 2011; Magallón et al., 2013). During the Late Carboniferous to the Late Triassic (311–212 mya), the five major gymnosperm lineages (cycads, ginkgos, cupressophytes, Pinaceae, and gnetophytes) separated from each other, making them much older than the earliest existing angiosperms (Magallón et al., 2013). In our study, on the basis of 29 shared protein‐coding genes, we estimated the divergence of the gymnosperms and angiosperms to have taken place ∼380 mya, which is consistent with the recent findings of Hohmann et al. (2018), who also used chloroplast data to infer the divergence of *Ginkgo biloba*. These authors found that the divergence of gymnosperms and angiosperms occurred an estimated 388 mya, which is in the same order of magnitude as earlier estimations of 360 (Clarke et al., 2011) and 330 (Magallón et al., 2013) mya.

One of the challenges faced in creating a gymnosperm phylogeny is the position of the Gnetales (Palmer et al., 2004), which remains unresolved despite much research effort. Our placement of Gnetales, according to analysis of 29 chloroplast protein‐coding genes, is consistent with the Gnetales–other gymnosperm hypothesis (Braukmann et al., 2009): we found that Gnetales was a sister to all other gymnosperms. Numerous phylogenomic studies have attempted to resolve phylogenetic position of Gnetales (De La Torre‐Bárcena et al., 2009; Cibrián‐Jaramillo et al., 2010; Zhong et al., 2010; Burleigh et al., 2012). Notably, most research has focused on concatenated protein‐coding nuclear genes (ESTs) and also supports the Gnetales–other gymnosperm hypothesis (De La Torre‐Bárcena et al., 2009; Cibrián‐Jaramillo et al., 2010; Lee et al., 2011), whereas the alternative Gnepine hypothesis is supported by analyses of plastome genes (Zhong et al., 2010; Wu, Wang et al., 2011). At present, however, neither the Gnetales–other seed plant hypothesis nor the Gnetales–other gymnosperm hypothesis is commonly accepted. One of the main reasons for this is the amount of missing data in the datasets used for phylogenomic analysis, which impedes phylogenetic inference (Roure et al., 2013).

Gymnosperms other than the Gnetales have also been investigated in several phylogenetic studies. The monotypic genus *Ginkgo*, for example, is the only survivor from the ginkgos for over at least 270 mya, and its systematic status has long been contentious (Wu et al., 2013). Some research indicates that *Ginkgo* is more similar to conifers than cycads, on the basis of comparative evolutionary analysis of spermatozoids (Norstog et al., 2004), or that it is intermediate between these two lineages, according to embryogenesis (Wang & Ran, 2014). However, several molecular phylogenetic studies based on one gene or a few genes favor *Ginkgo* as a sister of a clade of conifers and gnetophytes (Chaw et al., 2000; Mathews, 2009; Ran et al., 2010). Recent phylogenomic studies support a sister relationship between *Ginkgo* and the cycads (Cibrián‐Jaramillo et al., 2010; Finet et al., 2010; Xi et al., 2013), which is consistent with the shared morphological characteristics of the two, including the haustorial pollen tube (Friedman, 1993) and multiflagellated sperm (Ikeno & Hirase, 1897). Similarly, our results indicate that *Ginkgo* has a sister relationship with cycads.

Previously, plastome genes, noncoding regions, and nrDNA were used to infer the phylogenetic position of the cycads (Rai et al., 2003; Zgurski et al., 2008; Crisp & Cook, 2011). The basal location of *Cycas* and the division of cycads into two families, documented in the recent book by Osborne et al. (2012), have been confirmed by all these phylogenies. In other words, the genus *Cycas* is contained by the Cycadaceae, whereas the other nine genera comprise the Zamiaceae. Nevertheless, some intergeneric links within the Zamiaceae, especially the phylogenetic positions of *Stangeria*, *Dioon*, and *Bowenia*, remain unresolved, although most phylogenies have placed *Dioon* in a basal position (Rai et al., 2003; Chaw et al., 2005; Zgurski et al., 2008; Crisp & Cook, 2011). Recently, nuclear genes were used to reconstruct a comparatively large phylogeny of cycads in which *Dioon* diverged first, followed by *Bowenia* (in Zamiaceae), then an encephalartoid clade (*Encephalartos–Lepidozamia*–*Macrozamia*), which was a sister to a zamioid clade (Salas‐Leiva et al., 2013). Our findings, however, indicated that *Stangeria, Zamia*, and *Ceratozamia* diverged first, followed by *Dioon*, *Bowenia*, then the encephalartoid clade. Wang et al. (2000) generated the first molecular phylogeny of all 11 Pinaceae genera with chloroplast, mitochondrial, and nuclear genes; they discovered that the different gene trees were similar, with the exception of the phylogenetic placement of *Cathaya*, *Picea*, and *Pinus*. This supports the division of the pine family into two main groups: *Abies*–*Keteleeria*–*Nothotsuga*–*Tsuga*–*Pseudolarix*–(*Cedrus*) and *Cathaya*–*Picea*–*Pinus*–*Pseudotsuga*–*Larix*. Having conducted comparative chloroplast genomics, Lin et al. (2010) concluded that *Cedrus* was a sister to the clade of *Abies*–*Keteleeria*, and that *Cathaya* was more closely related to *Pinus* than *Picea*. However, it is noteworthy that *Cathaya* appears to be a hybrid of *Picea* and *Pinus*. Thus, *Abies* and *Kateleeria*, *Nothotsuga*, and *Pseudolarix* form clades with *Tsuga* to form a very similar clade. Within the Pinaceae, *Cathaya* is not closely linked to any other genera. In addition, *Sciadopitys* in Conifers II is the only member of its family and is not distantly related to other genera; it is found between theAraucariaceae–Podocarpaceae and Taxaceae. For Conifers II, prior molecular phylogenetic studies have repeatedly revealed interfamilial relationships: the Araucariaceae and Podocarpaceae diverged first, followed by the Sciadopityaceae, then the Taxaceae–Cephalotaxaceae, which is a sister to the Cupressaceae (Rai et al., 2008; Crisp & Cook, 2011; Burleigh et al., 2012, Yang et al., 2012). Similar relationships were observed in our study, in which the Podocarpaceae and Araucariaceae diverged first followed by the Sciadopityaceae, Taxaceae, and Cupressaceae.

## 5 CONCLUSIONS

Much effort has attempted to decode gymnosperm plastomes over the last two decades, which has considerably expanded the available plastomic data and given us a better picture of the evolution of gymnosperm plastomes. The elucidated plastomes of the five gymnosperm groups vary in their genome architecture, size, gene order, SSR, and IR evolution. The IRs have been lost in all conifers and exhibits several infrequent characteristics, such as size variation, genome rearrangements, diverse repeats, and disruptions of several conserved gene clusters. Despite considerable effort to determine gymnosperm plastomes at the genus or species level, certain families remain poorly sampled; therefore, detailed systematic phylogenetic studies are still lacking. The tree height between present day angiosperms and gymnosperms based on 29 shared genes was found to be 380 mya in this study. Our time‐calibrated plastid‐based phylogenomic tree provides a highly relevant framework for future comparative studies of gymnosperm evolution. Sequencing more plastomes and comparative analyses of these will provide more comprehensive insights into the evolution of gymnosperm plastomes.

## AUTHOR CONTRIBUTIONS

L., S.A, and A.L.K performed the analysis. R.J, Arif K, and Adil. K., performed the SSR and phylogenetic analyses. K.K and I.L. edited and drafted the manuscript.

## CONFLICTS OF INTEREST

The authors declare no conflict of interest.

## Supporting information




**Supplementary Figure S1**. Summary of gene losses and gains across gymnosperm plastomes.

**Supplementary Table S1**. Serial numbers representing the plastomes on a circular map (Circos)

**Supplementary Table S2**. Calibrations for relaxed molecular clock dating

## Data Availability

All the 167 plastomes are available at the NCBI database.
