# Supplementary material for: The dynamic history of gymnosperm plastomes: Insights from structural characterization, comparative analysis, phylogenomics, and time divergence
Source: Plant Genome. 2021 Sep 10;14(3):e20130. doi: 10.1002/tpg2.20130 (PMC12807257; doi:10.1002/tpg2.20130)
Supplement: Supplementary file 1 [file TPG2-14-e20130-s002.docx]

| Supplementary Table 2. Calibrations for relaxed molecular-clock dating | | | |  |
| --- | --- | --- | --- | --- |
|  |  |  |  |  |
| **Node** | **Phylogenetic placement** | **Fossil (reference)** | **Age (Ma)** | **Prior BEAST** |
| 1 | Seed plant crown | First 'modern' seeds with fused integuments [1, 2] | 325-385 | Normal mean 355 SD 15 |
| (root) |  |  |  |  |
| 2 | Angiosperm crown | Secondary based on the older estimate (without eudicot calibration) [3] | 228 (193-270) | LN offset 180 LM 3.8 LSD 0.5 |
| 3 | Gymnosperm crown | First Cordaitales (probably also stem conifers) [2, 4] | ~318 | LN offset 310 LM 1.5 LSD 0.5 |
| 4 | Cycad crown | *Crossozamia* [5] | ~270 | LN offset 251 LM 3 LSD 1 |
| 5 | Conifer crown | First Pinaceae-type cones [2] co-existent with Taxaceae-like fossils from sister group; consistent with estimate by Smith *et al.* [3] | ~225 | LN offset 225 LM 2 LSD 1 |
| 6 | *Bowenia* crown | *B.* spp. from early Eocene [6] | 49-55 | LN offset 45 LM 2 LSD 0.5 |
| 7 | *Lepidozamia* crown | *L. foveolata* from mid-Eocene [6] | 40.4-48.6 | LN offset 35 LM 2 LSD 0.5 |
| 8 | *Macrozamia* stem | *M. australis* from early Oligocene [6] | 28.4-33.9 | Uniform: min 28 max 355 |
| 9 | *Dioon* crown | *D.* sp. from Palaeocene [5] | 55.8-65.5 | LN offset 50 LM 2 LSD 0.5 |
| 10 | Pinaceae crown | Turonian cones [2, 7] | 89-93.5 | LN offset 90 LM 2 LSD 1 |
| 11 | Podocarpaceae-Cupressaceae s.l. | *Parasciadopitys* seed cones from lower middle Triassic [8] | 237-245 | Normal mean 241 SD 2 |
| 12 | Araucariaceae-Podocarpaceae | Co-existent mid-Jurassic fossils of both lineages [9, 10] | 160-175 | LN offset 155 LM 3 LSD 1 |
| 13 | *Sciadopitys*-Cupressaceae | Probable *Sciadopitys* stem fossils from Cenomanian [8] | 83.5-99.5 | LN offset 83.5 LM 3 LSD 2 |
| Gymnosperm classification follows Christenhusz *et al.* [11]. Stratigraphic dates follow Gradstein and Ogg [12]. Abbreviations: LN = lognormal LM = Log_e_(mean) LSD = Log_e_(standard deviation). | | | | |

**References**

1.	Doyle, J.A. (2006). Seed ferns and the origin of angiosperms. The Journal of the Torrey Botanical Society, 169-209.

2.	Won, H., and Renner, S.S. (2006). Dating dispersal and radiation in the gymnosperm Gnetum (Gnetales)—clock calibration when outgroup relationships are uncertain. Systematic biology *55*, 610-622.

3.	Smith, S.A., Beaulieu, J.M., and Donoghue, M.J. (2010). An uncorrelated relaxed-clock analysis suggests an earlier origin for flowering plants. Proceedings of the National Academy of Sciences *107*, 5897-5902.

4.	Magallón, S. (2010). Using fossils to break long branches in molecular dating: a comparison of relaxed clocks applied to the origin of angiosperms. Systematic biology *59*, 384-399.

5.	Hermsen, E.J., Taylor, T.N., Taylor, E.L., and Stevenson, D.W. (2006). Cataphylls of the Middle Triassic cycad Antarcticycas schopfii and new insights into cycad evolution. American Journal of Botany *93*, 724-738.

6.	Hill, R.S. (1998). Fossil evidence for the onset of xeromorphy and scleromorphy in Australian Proteaceae. Australian Systematic Botany *11*, 391-400.

7.	Gandolfo, M., Nixon, K., and Crepet, W. (2001). Turonian Pinaceae of the Raritan Formation, New Jersey. Plant Systematics and Evolution *226*, 187-203.

8.	Stockey, R.A., Kvacek, J., Hill, R.S., Rothwell, G.W., and Kvacek, Z. (2005). The fossil record of Cupressaceae s. lat. A monograph of Cupressaceae and Sciadopitys *54*, 68.

9.	Hill, R.S., and Brodribb, T. (1999). Turner Review No. 2-Southern conifers in time and space. Australian Journal of Botany *47*, 639-696.

10.	Biffin, E., Hill, R.S., and Lowe, A.J. (2010). Did kauri (Agathis: Araucariaceae) really survive the Oligocene drowning of New Zealand? Systematic biology *59*, 594-602.

11.	Christenhusz, M.J., Reveal, J.L., Farjon, A., Gardner, M.F., Mill, R.R., and Chase, M.W. (2011). A new classification and linear sequence of extant gymnosperms. Phytotaxa *19*, 55-70.

12.	Gradstein, F., and Ogg, J. (2004). Geologic time scale 2004–why, how, and where next! Lethaia *37*, 175-181.
